# A proteomic study of mitotic phase-specific interactors of EB1 reveals a role for SXIP-mediated protein interactions in anaphase onset

**DOI:** 10.1242/bio.201410413

**Published:** 2015-01-16

**Authors:** Naoka Tamura, Judith E. Simon, Arnab Nayak, Rajesh Shenoy, Noriko Hiroi, Viviane Boilot, Akira Funahashi, Viji M. Draviam

**Affiliations:** 1 Department of Genetics, University of Cambridge, Cambridge, UK; 2 Present address: European Research Institute for the Biology of Ageing, University of Groningen, Groningen, Netherlands.; 3 Present address: Institute for Biochemistry II, Goethe University Frankfurt am Main, Germany.; 4 Keio University, Yokohama, Japan

**Keywords:** Cell cortex, Kinetochore, Microtubule, Mitosis, Plus-end

## Abstract

Microtubules execute diverse mitotic events that are spatially and temporally separated; the underlying regulation is poorly understood. By combining drug treatments, large-scale immunoprecipitation and mass spectrometry, we report the first comprehensive map of mitotic phase-specific protein interactions of the microtubule-end binding protein, EB1. EB1 interacts with some, but not all, of its partners throughout mitosis. We show that the interaction of EB1 with Astrin-SKAP complex, a key regulator of chromosome segregation, is enhanced during prometaphase, compared to anaphase. We find that EB1 and EB3, another EB family member, can interact directly with SKAP, in an SXIP-motif dependent manner. Using an SXIP defective mutant that cannot interact with EB, we uncover two distinct pools of SKAP at spindle microtubules and kinetochores. We demonstrate the importance of SKAP's SXIP-motif in controlling microtubule growth rates and anaphase onset, without grossly disrupting spindle function. Thus, we provide the first comprehensive map of temporal changes in EB1 interactors during mitosis and highlight the importance of EB protein interactions in ensuring normal mitosis.

## INTRODUCTION

During mitosis, microtubules play a crucial role in multiple concurrent events: in early mitosis, microtubules assemble a bipolar spindle, capture chromosomes at specialized sites called kinetochores, power chromosome movements and rotate the bulky spindle apparatus towards a predetermined axis. Following the completion of chromosome congression in metaphase, cells initiate anaphase – a phase when microtubules pull and separate chromatids apart, establish the plane for cleavage furrow formation and facilitate anaphase cell elongation. How mitotic microtubules are controlled to coordinate such diverse tasks, in a spatially and temporally defined manner, is a fascinating and poorly understood biological problem.

The microtubule-end binding protein EB1 forms a comet-like structure specifically at the plus-ends of growing microtubules (Mimori-Kiyosue et al., 2000) and a crescent-like structure at the kinetochore following microtubule attachment (Tirnauer et al., 2002). Although members of the EB family, EB1 and EB3, act redundantly to regulate microtubule growth in interphase cells (Komarova et al., 2009; Komarova et al., 2005), they control diverse spatially and temporally separated mitotic events, including kinetochore alignment (Draviam et al., 2006; Green et al., 2005), spindle orientation (Brüning-Richardson et al., 2011; Draviam et al., 2006; Ferreira et al., 2013; Green et al., 2005; Toyoshima and Nishida, 2007) and post-cytokinetic cell spreading (Ferreira et al., 2013). However, the underlying molecular mechanisms are not understood.

Several EB1 or EB3 interacting proteins have been identified using large-scale immunoprecipitations from asynchronous cell populations (Berrueta et al., 1999; Geraldo et al., 2008; Gu et al., 2006; Meireles et al., 2011; Rogers et al., 2004; Schrøder et al., 2011). In interphase, EB1 interacts with several partners to modulate interphase microtubule plus-end function (reviewed in Akhmanova and Steinmetz, 2008; Jiang et al., 2012). A clear molecular understanding of EB1's mitotic function requires a comprehensive list of EB-interactors from temporally separated distinct phases of mitosis and this is currently lacking.

EB proteins interact with several partner proteins bearing either a CAP-Gly rich domain or an S/T-X-I/L-P motif (referred as ‘SXIP-motif’) (reviewed in Akhmanova and Steinmetz, 2010; Tamura and Draviam, 2012). While residues adjacent to the SXIP motif could render further specificity for EB1 protein interactions (Buey et al., 2011; Honnappa et al., 2009; Jiang et al., 2012), phosphorylation around EB1 binding motifs is reported to be a crucial determinant of EB1 protein interactions during both interphase and mitosis (Honnappa et al., 2005; Smyth et al., 2012; Kumar et al., 2012; Kumar et al., 2009; van der Vaart et al., 2011; Watanabe et al., 2009; Wittmann and Waterman-Storer, 2005; Zimniak et al., 2009). However, it is not known if disruption of EB protein interaction would modulate microtubule dynamics during mitosis as it does during interphase (reviewed in Tamura and Draviam, 2012).

To determine how microtubule plus-ends execute distinct mitosis phase-specific events, we searched for EB1 interactors from two distinct phases of mitosis using large-scale immunoprecipitation and mass spectrometry. Our proteome-wide effort revealed the spindle and kinetochore associated protein, SKAP, as a mitotic phase dependent interactor of EB1. We show that SKAP's SXIP-motif is essential for interacting with both EB1 and EB3. Using an SXIP defective mutant, we show that the SXIP-motif is important for proper mitotic microtubule growth rates and SKAP overexpression induced delay in anaphase onset. Our findings show that an excess of SKAP-EB interaction can result in an anaphase onset delay, without grossly affecting other microtubule-mediated functions such as bipolar assembly or chromosome congression. We present a model wherein finely regulated interaction of microtubule plus-end complexes is a key rate-limiting factor for determining the onset of anaphase.

## RESULTS

### Mitotic phase determined interactions of EB1

To identify microtubule plus-end bound complexes from distinct phases of mitosis, we performed large-scale immunoprecipitation of Flag-tagged EB1 (Flag-EB1) from prometaphase and anaphase cell lysates. Flag-tagged Nuf2 (Flag-Nuf2) was used as a bait control because the human Ndc80-Nuf2 complex is a core-kinetochore protein which was shown using Electron Microscopy to interact with microtubule walls (Cheeseman et al., 2006) and to associate with disassembling microtubule-ends (Umbreit et al., 2012), as opposed to EB1 that binds selectively to growing microtubule-ends (Komarova et al., 2009; Mimori-Kiyosue et al., 2000).

**Fig. 1.**
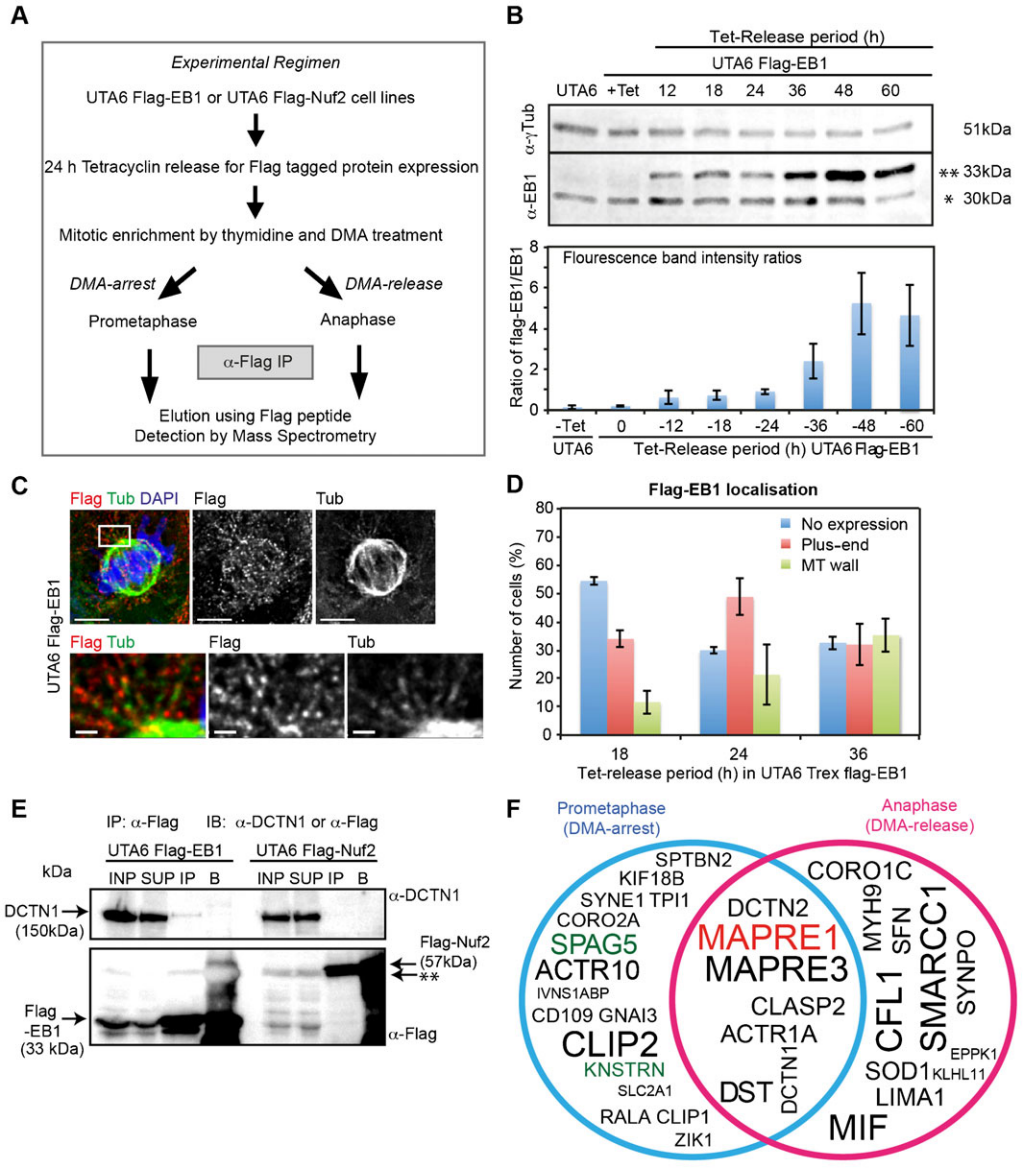
Mitotic phase-specific interactions of the plus-end protein, EB1. (A) Protocol used to identify mitotic phase-specific interactors of EB1 as detailed in Materials and Methods. (B) Fluorescence immunoblot (upper panel) and intensity graph (lower panel) levels of Flag-EB1 (**) relative to endogenous EB1 (*) following Tetracycline release. UTA6 or UTA6 Flag-EB1 lysates were immunoblotted with α-EB1 and α-γTubulin (loading control) antibodies. (C) Immunofluorescence images of UTA6 Flag-EB1 cells released from Tetracycline for 24 h and immunostained with α-Flag and α-Tubulin (Tub) antibodies and co-stained with DAPI for DNA. Lower panels show magnified images of area boxed in white. Scale: 5 µm (upper panel) and 1 µm (lower panel). (D) Graph of percentage of UTA6 Flag-EB1 cells released from Tetracycline (Tet) for varying periods, immunostained as in (C) and counted for cells with Flag-EB1 signals at microtubule plus-end (Plus-end), microtubule (MT) wall or no Flag-EB1 signals (No expression). Error bar represents SEM from three independent experiments. (E) Fluorescent immunoblots of α-Flag immunoprecipitates from lysates of UTA6 Flag-EB1 and UTA6 Flag-Nuf2, probed with anti-p150 (DCTN1) and anti-Flag antibodies as indicated. Cell lysates (INP), supernatant (SUP) following immunoprecipitation, Flag-peptide eluted immunoprecipitate (IP) and beads fraction (B) are loaded. ** indicates non-specific band. (F) Venn diagram showing EB1 interactors from DMA-arrest (Blue circle), -release (Pink circle) or both (overlapped region) conditions. Diagram excludes proteins found in Flag-Nuf2 IP (corresponding to each batch of DMA treatment) and includes proteins found in Flag-EB1 IPs at least twice. Font size in each area of the circle reflects reproducibility across repeats. Bait (MAPRE1/EB1) and Astrin(SPAG5)-SKAP(KNSTRN) are highlighted red and green, respectively.

For large-scale enrichment of mitotic cells, we treated cells with DMA, an Eg5 inhibitor that induces monopolar spindles and arrests cells in prometaphase, and then performed mitotic shakeoff for isolating rounded-up prometaphase cells; for anaphase cells, we washed the rounded-up cells to remove the inhibitor and synchronously released the cells into anaphase (Fig. 1A). DMA treatment of UTA6-Flag-EB1 cells allowed the enrichment of mitotic cells to approximately 50% of the total cell population (data not shown). As expected from our previous studies of monopolar to bipolar spindle conversion (Shrestha et al., 2014), prometaphase UTA6 cells were predominantly in anaphase following a 45 min release from DMA treatment, as confirmed using microscopy of UTA6 cultures (supplementary material Fig. S1A). Thus, the DMA treatment and mitotic shake-off protocol allowed us to obtain high-quality lysates of cells from two different mitotic phases.

To exclude immunoprecipitation artefacts, we performed five quality control steps: First, we ensured that Flag-EB1 was expressed at levels comparable to endogenous EB1 by modulating Tetracycline-release induced protein expression conditions (Fig. 1B). Second, we confirmed that the localization of Flag-EB1 was restricted to the plus-ends of microtubules (Fig. 1C,D). Third, we used Flag peptides to specifically elute Flag-EB1 and associated complexes. Fourth, to exclude contaminants, we compared mass spectrometry data of immunoprecipitates from UTA6-Flag-EB1 and UTA6-Flag-Nuf2 cells and excluded all common interactors. Although we may lose their common interactors at the kinetochore, we were not concerned because first, Gene Ontology (GO) analysis of the excluded common interactors did not show any enrichment for microtubule plus-end complexes and second, we did not find EB proteins in any of the Nuf2 immunoprecipitates (data not shown). Finally, to ensure that the protein candidates identified are true hits in the proteomic database, we only considered proteins that were identified using at least two distinct peptides. We first confirmed that these five stringent steps allowed us to immunoprecipitate established EB1 interactors, such as p150 (DCTN1), specifically from UTA6-Flag-EB1 but not UTA6-Flag-Nuf2 cell line (Fig. 1E).

For building the EB1-interacting proteome from distinct mitotic phases, we extracted from our mass spectrometry data only the specific interactors of EB1 that were reproducibly found in at least 2 repeats of Flag-EB1 immunoprecipitations, and never in any of the repeats of Flag-Nuf2 immunoprecipitations (Tables 1, 2). To exclude artefacts, we considered only those proteins where at least two distinct peptides could be recovered. Some interactors of EB1 are common to both prometaphase and anaphase; however, many others are specific to either prometaphase or anaphase (Fig. 1F; supplementary material Fig. 1B). We then compared our list of mitotic phase-specific flag-EB1 interactors against previously obtained list of GST-EB1 interactors from asynchronous human cell cultures that should include a small proportion of mitotic cells (Jiang et al., 2012). This comparison study showed that while nearly half of our hits could be observed in asynchronous conditions as well, at least 40% of the hits could be visualized only in conditions that enrich for mitotic cells (Tables 1, 2).

**Table 1.**
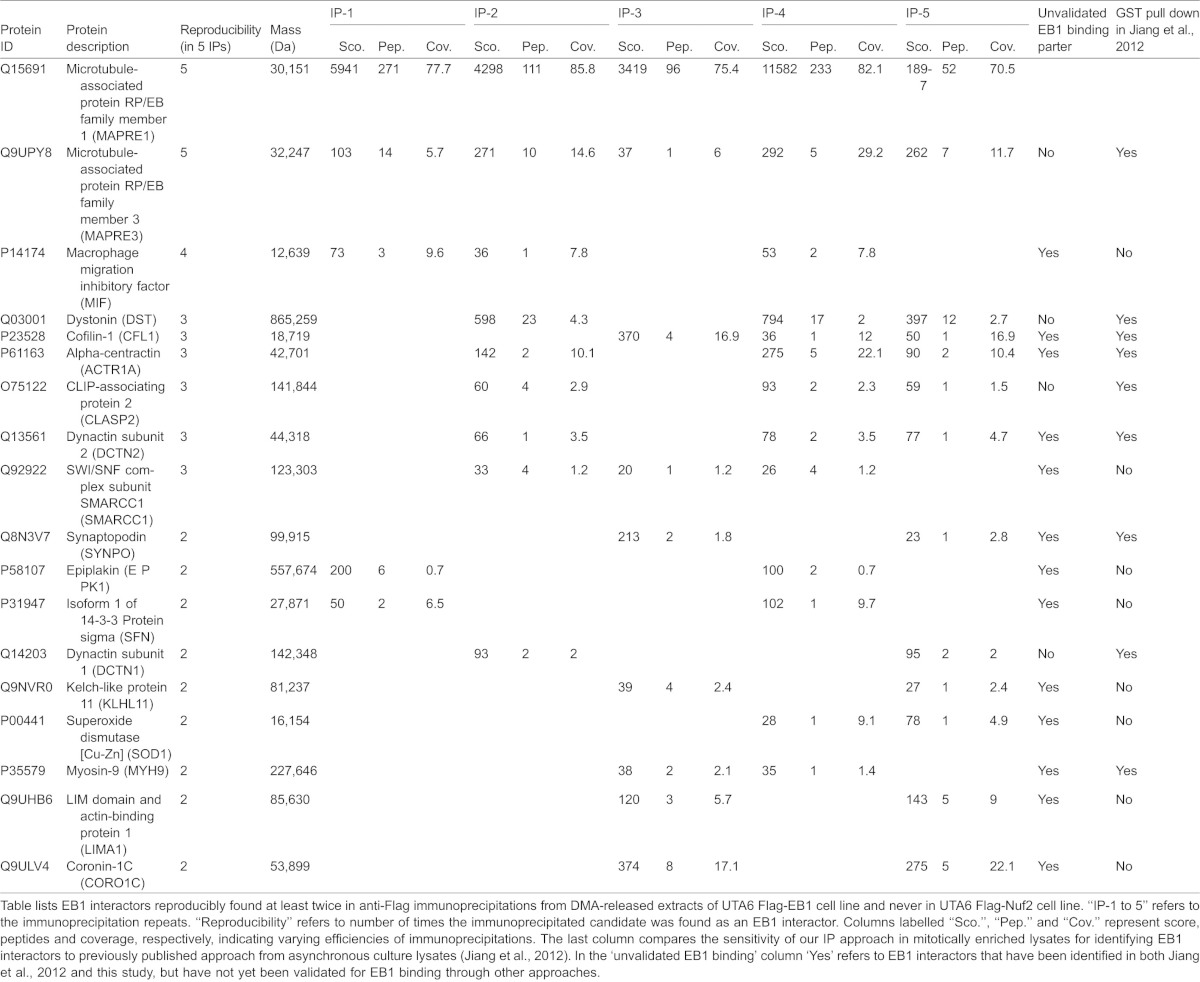
Summary of mass spectrometry based identification of EB1 interactors in DMA-released mitotic cell extracts

**Table 2.**
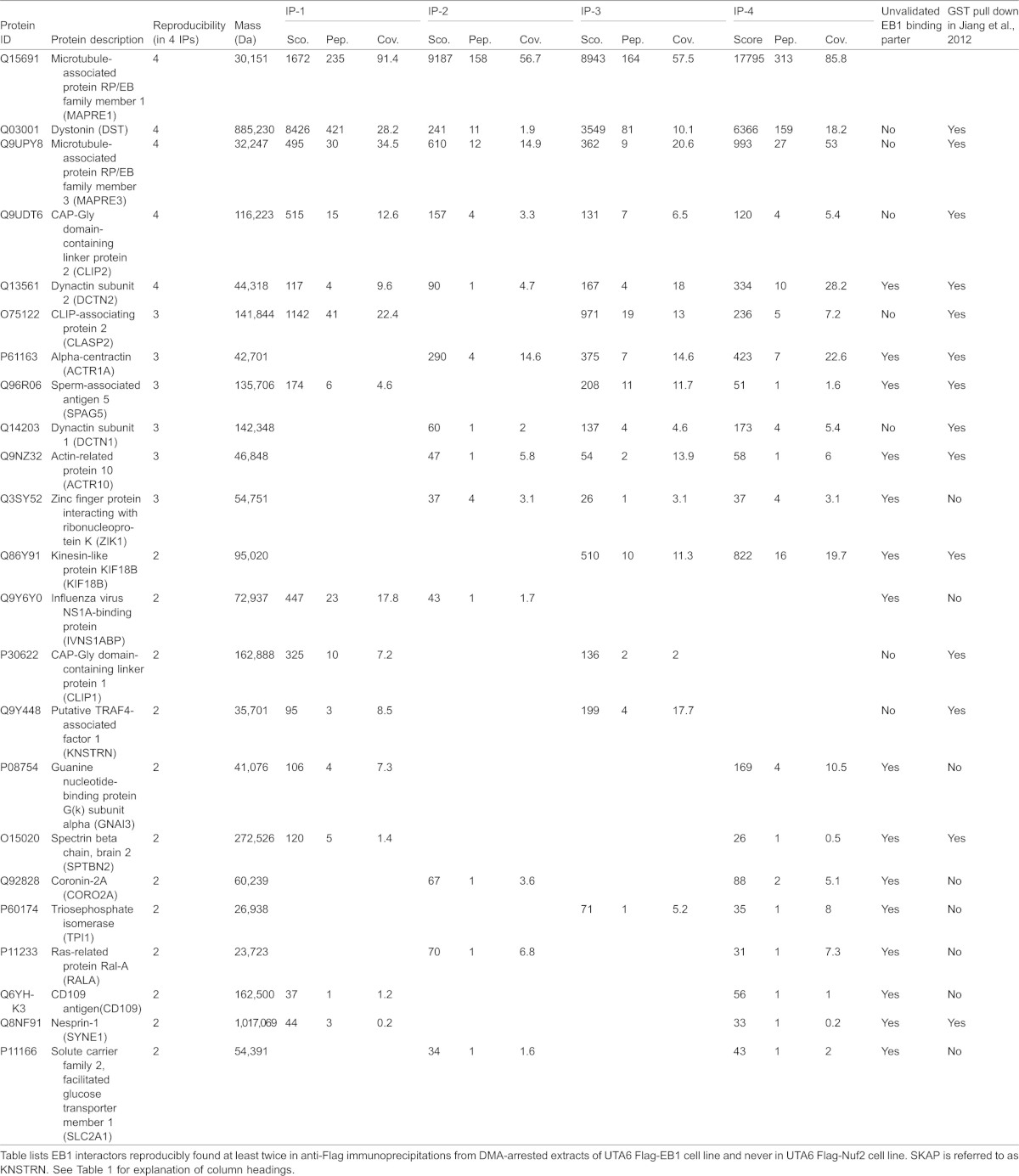
Summary of mass spectrometry based identification of EB1 interactors in DMA-arrested released mitotic cell extracts

As a strong evidence of success in our proteome-wide search for mitosis phase-specific plus-end complexes, we found the plus-end tracking kinesin, Kif18b (KIF18B) from prometaphase, but not anaphase, extracts of UTA6-Flag-EB1 cells (Fig. 1F; supplementary material Fig. 1B). This is consistent with previous studies showing the plus-end localization of Kif18B in early mitosis but not anaphase (Lee et al., 2010; Stout et al., 2011). Thus, our data presents the first comprehensive map of mitotic phase-specific interactors of EB1.

### Astrin-SKAP complex is a mitotic phase-specific interactor of EB1

Among the protein complexes that were immunoprecipitated with EB1, the Astrin (SPAG5)-SKAP complex, a known regulator of spindle and kinetochore function (Dunsch et al., 2011; Gruber et al., 2002; Schmidt et al., 2010) and a marker of kinetochores bearing mature attachments to microtubule-ends (Shrestha and Draviam, 2013), was reproducibly observed in Flag-EB1 immunoprecipitates from prometaphase cells but not anaphase cells (Fig. 1F; supplementary material Fig. 1B).

**Fig. 2.**
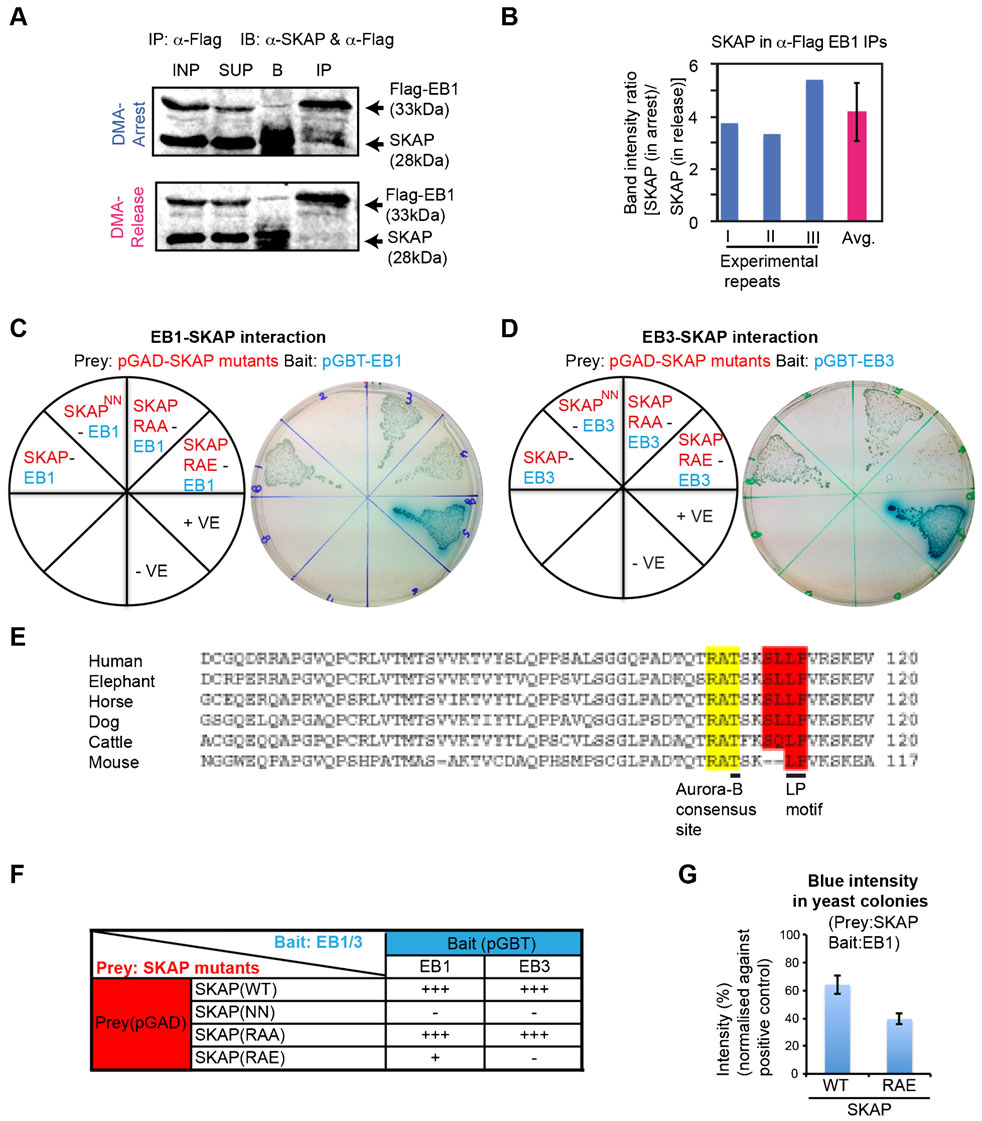
Astrin-SKAP complex interacts with EB1 in a mitotic phase-specific manner. (A) Immunoblots showing increased SKAP in α-Flag immunoprecipitates (IP) from lysates of prometaphase (DMA-arrest) compared to anaphase (DMA-release) UTA6 Flag-EB1 cells. Immunoblots probed with α-Flag and α-SKAP antibodies in two fluorescent channels (merge presented). Cell lysates (INP), Supernatant (SUP) following immunoprecipitation and beads fraction (B) are loaded. (B) Graph showing reproducibility of SKAP intensity ratios from immunoblots (as in A) of α-Flag-EB1 immunoprecipitates from DMA-arrest (prometaphase) and -release (anaphase) cell lysates. Ratio from three independent repeats, their average (avg.) and SD values (error bars) are shown. (C,D) Yeast two-hybrid study of strains bearing prey and bait protein expression vectors as indicated in red and blue on the pie chart (left). Positive protein-protein interaction was assessed through the activation of the *lacZ* reporter gene, which was demonstrated by the formation of blue colonies on plates containing X-Gal. Photographs (right) of colonies in (C) show blue colour development indicating interaction between EB1 and SKAP (SKAP::EB1), EB1 and phospho-dead SKAP mutant (SKAP(RAA)::EB1). No blue colour development indicates no interaction between EB1 and SKAP (NN) mutant (SKAP(NN)::EB1). Blue colour development is reduced indicating weaker interaction between EB1 and phospho-mimetic SKAP mutant (SKAP(RAE)::EB1). The interaction between SV40-p53 and SV40-Laminin were used as positive (+ve) and negative (-ve) controls, respectively. In (D), photographs (right) of colonies show blue colour development indicating positive interaction between EB3 and SKAP (SKAP::EB3), EB3 and phospho-dead SKAP mutant (SKAP(RAA)::EB3) and no blue colour development indicating no interaction between EB3 and SKAP (NN) mutant (SKAP(NN)::EB3) and EB3 and phospho-mimetic SKAP mutant (SKAP(RAE)::EB3). (E) Sequence alignment using ClustalW2 showing evolutionary conservation of the S/T-X-I/L-P domain of SKAP in mammals (Human: Q9Y448; Mouse: Q9D9Z1; Horse: F6T184; Cattle: E1BJ69; Dog: E2RST7; Elephant: G3TLE9). Yellow and Red highlights mark evolutionarily conserved Aurora-B consensus site and S/T-X-I/L-P motifs, respectively. (F) Summary table of interaction between SKAP mutants with either EB1 or EB3. + and − refer to positive and no interaction, respectively. +++ and + refer to strong and weak interaction, respectively. (G) Graph of blue intensity of yeast colonies show a reduction in interaction between EB1 and SKAP(RAE) mutant compared to EB1 and SKAP. Percentages of blue intensity values were obtained by normalising against intensities of colonies in positive controls (+ve) and are batch controlled. Error bars indicate SD values.

Interaction between EB1 and SKAP is known (Wang et al., 2012), but whether the interaction is subjected to mitotic phase dependent regulation was not known. Therefore, to confirm our findings from the proteome-wide study, we investigated if EB1 interacts with SKAP in a mitotic phase-specific manner using quantitative fluorescent immunoblotting. This allowed us to quantify and compare the amount of SKAP across three independent repeats of Flag-EB1 immunoprecipitations from prometaphase and anaphase cells (Fig. 2A,B). Anti-Flag immunoprecipitations from UTA6 Flag-EB1 cell lysates reproducibly showed that the interaction of EB1 with SKAP was on average four-fold higher in prometaphase lysates with high Cyclin-B levels, compared to anaphase lysates with low Cyclin-B levels (Fig. 2B; supplementary material Fig. S1C). Because Cyclin-B starts degrading at the end of metaphase and continues into anaphase but is not degraded in prometaphase (Clute and Pines, 1999), the difference in Cyclin-B levels further confirms the successful separation of the two mitotic phases. These data reveal the Astrin-SKAP complex as a mitotic phase determined interactor of EB1.

### Both EB1 and EB3 interact directly with SKAP of the Astrin-SKAP complex

EB1 and EB3 have a highly conserved EB homology domain (reviewed in Tamura and Draviam, 2012). However, during mitosis, the two proteins play non-redundant roles. EB1 is required for spindle positioning in metaphase and chromosome segregation in anaphase (Draviam et al., 2006). In contrast, EB3, but not EB1, is required for spindle positioning in anaphase (Ferreira et al., 2013). It is not known if both EB1 and EB3 are capable of interacting with SKAP. Although many interphase partners of EB1 can redundantly bind to EB3 as well (Bu and Su, 2003; Komarova et al., 2005; Mimori-Kiyosue et al., 2005; van der Vaart et al., 2011), it is not known if mitotic interactors have similar redundancy, and this is important to study because EB1 and EB3 regulate non-redundant mitotic functions (Ferreira et al., 2013).

To test if both EB1 and EB3 are capable of interacting with SKAP, we used yeast two-hybrid (Y2H) assays (Fig. 2C,D). For control studies, we mutated LP into NN in the S/T-X-I/L-P motif since similar mutations have been shown to abrogate the interaction between EB and its partners (Honnappa et al., 2009; Jiang et al., 2012). Y2H studies showed that EB1 interacts with SKAP Wild-Type (WT) but not the SKAP(NN) mutant with a defective SXIP-motif (Fig. 2C), consistent with previous report (Wang et al., 2012), confirming the role of the SXIP-motif in mediating SKAP-EB1 interaction. In addition, we found that similar to EB1, EB3 can also interact directly with SKAP and this interaction is also dependent on SKAP's SXIP-motif (Fig. 2D). We conclude that both EB1 and EB3 are capable of interacting with SKAP, in an SXIP-motif dependent manner. Thus, SKAP joins a family of proteins that are capable of interacting with either EB1 or EB3 (Bu and Su, 2003; Komarova et al., 2005; Mimori-Kiyosue et al., 2005; van der Vaart et al., 2011), unlike others that selectively interact with only one of the EB proteins (Goldspink et al., 2013; Straube and Merdes, 2007).

We next tested if Astrin, the other member of the Astrin-SKAP complex directly interacted with EB1, since Astrin is also immunoprecipitated with Flag-EB1 in a mitosis phase-specific manner (Tables 1, 2). Our Y2H studies showed that Astrin did not interact with EB1 although as expected Astrin interacted with SKAP (supplementary material Fig. S2). This shows that EB1 interacts specifically with SKAP of the Astrin-SKAP complex.

An evolutionarily conserved Aurora B-consensus site exists proximal to the SXIP-motif of SKAP (Fig. 2E). Previous studies have shown that the interaction between EB and its partners can be negatively regulated by phosphorylation close to the SXIP-motif (Buey et al., 2012) and such negative regulation has been reported during mitosis (Kumar et al., 2012; Zimniak et al., 2009). Moreover, Aurora-B is known to negatively regulate SKAP recruitment to kinetochores (Schmidt et al., 2010). Therefore, we investigated if SKAP-EB interaction is controlled by phosphorylation of the Aurora-B consensus site proximal to the SXIP-motif using either non-phosphorylatable (phospho-dead) or phospho-mimetic mutants by mutating RAT^108^ into RAA or RAE, respectively. Y2H studies showed that phospho-mimetic mutation of SKAP at T108 (RAT to RAE) significantly reduces SKAP interaction with EB1 (Fig. 2C,G) and completely abolishes SKAP interaction with EB3 (Fig. 2D,F). In contrast, the SKAP (RAT to RAA) mutant was able to interact with both EB1 and EB3 (Fig. 2C,D), showing the role of charged residues in modulating EB-SKAP interactions. Thus, in addition to the SXIP-motif, electrostatic interactions surrounding the SXIP-motif are critical for SKAP-EB interaction.

### SXIP-motif is essential for SKAP recruitment to spindle microtubules but not kinetochores

The functional significance of SKAP-EB interaction *in vivo* is not known, although *in vitro* studies show SKAP's interaction with EB1 to be important for its microtubule plus-end loading (Wang et al., 2012).

**Fig. 3.**
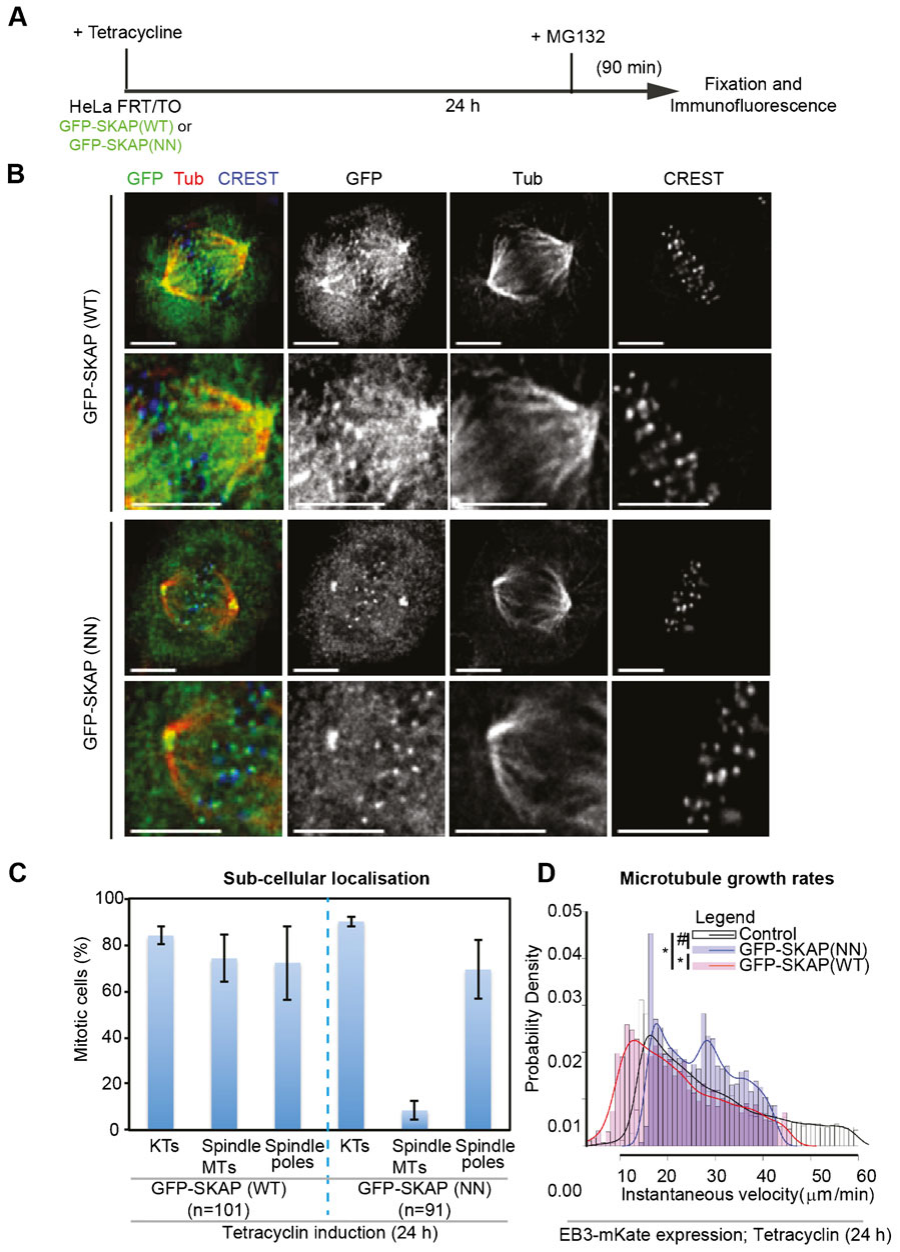
The ‘SXIP-motif’ of SKAP is required for SKAP localization at spindle microtubules but not kinetochores. (A) Schematic describing drug treatment regimen: HeLa FRT/TO cell lines were treated with Tetracycline for 24 h and then exposed to MG132 for 90 min prior to fixation. (B) Representative immunofluorescence images of HeLa FRT/TO cell line treated as in (A), expressing either GFP-SKAP(WT) or GFP-SKAP(NN) mutant. Cells were immunostained with α-GFP and α-Tubulin (Tub) antibodies and CREST antisera. Scale bar: 5 µm. Lower panels show magnified images. (C) Graph shows percentage of mitotic HeLa FRT/TO cells expressing either GFP-SKAP (WT) or GFP-SKAP (NN) mutant displaying SKAP localization at kinetochores (KTs), spindle microtubules (Spindle MTs) or Spindle poles. Scoring was based on SKAP enrichment or the lack of SKAP enrichment at specific subcellular sites. n refers to number of cells. Cells were treated as in (A). Error bars indicate SEM values across three independent repeats. (D) Probability density distribution plots for comparing the distribution of average values for instantaneous velocities of EB3 comets in cells expressing EB3-mKate either alone (Control) or together with GFP-SKAP(WT) or GFP-SKAP(NN) mutant. Values were obtained using plus-tip tracker software and outliers greater than 2×SD from the peak average values are excluded in the plot. Curves represent smoothened values of the bar plot values presented. At least four cells and 3300 comets were analyzed for each of the three conditions. Non-overlapping peak values between control and SKAP(WT), and SKAP(WT) and SKAP(NN) signify statistically significant differences (*) as confirmed by p<0.01 using the Wilcoxon rank sum test. Overlapping peak values between SKAP(NN) and Control signify statistically insignificant differences (#) as confirmed by p>0.01 using the Wilcoxon rank sum test.

To address the role of SKAP-EB interaction *in vivo*, we disrupted the interaction in cells using the SKAP(NN) mutant. We established HeLa FRT/TO cell lines that conditionally expressed either GFP-tagged SKAP (NN) mutant or (HeLa^GFP-SKAP(NN)^) or GFP-tagged SKAP Wild-Type (HeLa^GFP-SKAP(WT)^) in the presence of Tetracycline. Using immunofluorescence, we first analysed SKAP localization at kinetochore, spindle poles and spindle microtubules in cells treated with MG132, a proteasome inhibitor that arrests cells in metaphase (Fig. 3A). Following MG132 treatment, cells expressing GFP-SKAP(WT) or GFP-SKAP(NN) mutant displayed congressed chromosomes and both proteins localized normally to spindle poles and kinetochores (Fig. 3B,C). However, fluorescence intensity of GFP-SKAP(NN) mutant on spindle microtubules was much reduced compared to GFP-SKAP(WT) (Fig. 3B,C), revealing the role of SXIP-motif in localizing SKAP onto spindle microtubules.

We then measured microtubule growth using EB3 – a marker of growing microtubule-ends (Bu and Su, 2001; Nakagawa et al., 2000) – in live-cells co-expressing EB3-mKate and either GFP-SKAP(WT) or GFP-SKAP(NN) mutant. We assessed the instantaneous growth velocities of EB3-mKate comets automatically using the plus-tip tracker software (Matov et al., 2010). Microtubule growth velocities of EB3 comets in mitotic cells expressing EB3-mKate alone (average peak values from plus-tip tracker data: Control: 15 µm/min; n_comets_ = 17,923) was comparable to previously reported values for EB3 (Sironi et al., 2011) or EB1 (Corrigan et al., 2013) growth rates. However, cells over-expressing GFP-SKAP(WT), showed a significant reduction in microtubule growth rates compared to control cells (GFP-SKAP(WT): 12 µm/min; n_comets_ = 3347). Slightly higher average peak values of microtubule growth rates were observed in GFP-SKAP(NN) expressing cells (GFP-SKAP(NN) cells: 17 µm/min; n_comets_ = 4582), compared to controls. However, the overall distribution of growth velocities was significantly different between control and GFP-SKAP(WT) expressing cells, but not GFP-SKAP(NN) mutant expressing cells (Fig. 3D). We conclude that the regulation of SKAP-EB interaction is important for maintaining normal microtubule growth velocity during mitosis.

Collectively, these data shed first insight into the existence of two distinct pools of SKAP: a spindle microtubule associated pool that influences microtubule growth in an SXIP-motif dependent manner and a kinetochore bound pool that binds to congressed kinetochores in an SXIP-motif independent manner.

### SKAP overexpression delays anaphase onset, in an SXIP-motif dependent manner

**Fig. 4.**
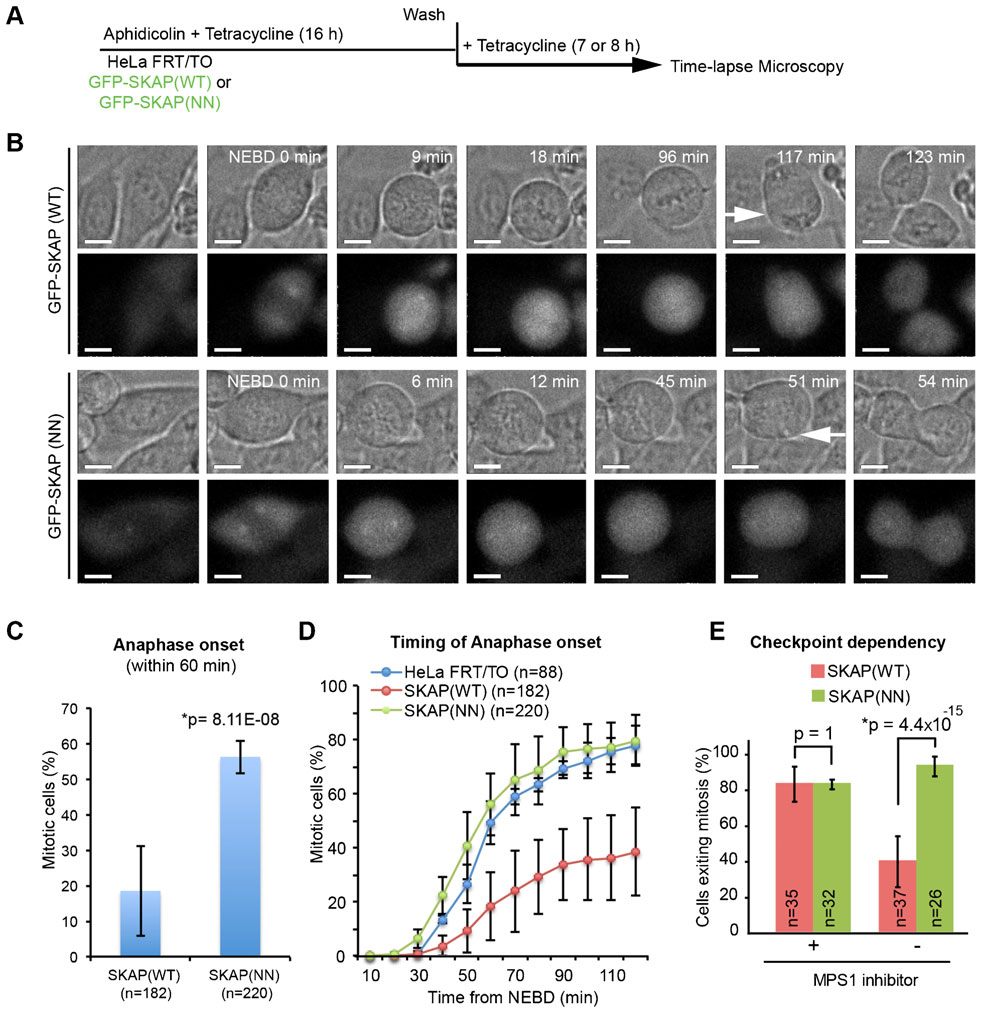
SKAP overexpression delays anaphase onset in an ‘SXIP-motif’ dependent manner. (A) Schematic describing drug treatment regimen: HeLa FRT/TO cells were treated with Tetracycline and synchronised using aphidicolin treatment for 16 h and then released from aphidicolin for 7 h prior to filming. (B) Time-lapse images of HeLa FRT/TO cells expressing GFP-SKAP (WT) or GFP-SKAP (NN) mutant. DIC images (top panels) show rounding up of cells, metaphase plate and anaphase (white arrows). Fluorescent images (bottom panels) show GFP signals. Cells were treated as in (A). Scale bar: 10 µm. (C) Graph of percentage of HeLa FRT/TO cells expressing GFP-SKAP (WT) or GFP-SKAP (NN) mutant, which initiated anaphase within 60 min from NEBD (nuclear envelope break down). NEBD was assessed using loss of exclusion of GFP-SKAP signal in nucleus and anaphase onset was assessed using anaphase cell elongation. Error bars indicate SEM values. *Statistical significance. p-value was calculated using proportion test. n indicates number of cells analysed. (D) Cumulative frequency plots of anaphase times in HeLa FRT/TO parental cells and HeLa FRT/TO cell lines expressing either GFP-SKAP(WT) or GFP-SKAP(NN) mutant. Cells that initiated and exited mitosis within 2 h were included. Average values were obtained from 3 independent experimental repeats of GFP-SKAP expressing cells and 2 independent experimental repeats of HeLa FRT/TO parental cells. Error bars indicate SEM values. (E) Graph of percentage of mitotic cells expressing either GFP-SKAP (WT) or GFP-SKAP (NN) mutant that initiated anaphase in the presence (+) or absence (-) of MPS1 inhibitor (400 nM NMS-P715) during 4 h of time-lapse imaging. Inhibitor was added just before time-lapse imaging session. Error bars show SD values and p-values were obtained using proportion test. *Statistically significant difference.

We next investigated if SKAP-EB interaction is important for mitotic progression using time-lapse microscopy (Fig. 4A). An interesting difference emerged between cells overexpressing SKAP(WT) or SKAP(NN) mutant. Consistent with a previous report of metaphase arrest in SKAP over-expressing cells (Dunsch et al., 2011), our time-lapse studies showed a clear metaphase arrest and delay in anaphase onset in cells overexpressing GFP-SKAP(WT) (Fig. 4B,C). Strikingly, however, cells overexpressing the GFP-SKAP(NN) mutant did not display any delay in anaphase onset, compared to the parental HeLa FRT/TO cell line (Fig. 4D), showing the anaphase delay to be SXIP-motif dependent. We confirmed that this striking difference in anaphase onset times between cells expressing SKAP(WT) or SKAP(NN) is not due to a difference in the amount of GFP-tagged SKAP(WT) and SKAP(NN) (supplementary material Fig. S3A,B). Because SKAP(WT) overexpressing but not SKAP(NN) cells display a prominent delay in anaphase onset despite normal chromosome congression and bipolar spindle assembly (Fig. 3B), we conclude that excessive SKAP-EB interaction delays anaphase onset without grossly disrupting other microtubule-mediated mitotic functions.

SKAP overexpression induced delay in anaphase onset could arise from either a biochemical inability in transitioning from metaphase to anaphase because of APC/C inactivation or a physical inability in pulling and segregating sister chromatids apart. To test if the delay in anaphase induced by SKAP overexpression is due to a delay in APC/C activation, we performed time-lapse microscopy of cells expressing either GFP-SKAP(WT) or GFP-SKAP(NN) mutant in the presence of NMS-P715, an inhibitor of MPS1 kinase that is required for kinetochore bound checkpoint signaling and subsequent Mitotic Checkpoint Complex assembly (Tipton et al., 2013; Zich et al., 2012). Around 80% of metaphase cells expressing GFP-SKAP(WT) or GFP-SKAP(NN) mutant were observed to initiate anaphase onset within 50 min of treating cells with MPS1 inhibitor (Fig. 4E). These data show that SKAP overexpression induced delay in anaphase is dependent on the spindle assembly checkpoint-induced inhibition of the APC/C. Thus, SKAP overexpression delays anaphase by inhibiting APC/C activation, in an SXIP-motif dependent manner.

Although overexpression studies are difficult for functional dissection, we find that the SKAP (SXIP) mutant lacks the SKAP (WT) overexpression induced phenotype. This suggests that there is a fine balance in SXIP mediated interactions and upsetting this balance can delay anaphase onset. This data obtained through SKAP overexpression studies is clinically relevant because the protein is overexpressed in breast carcinomas (Wright and Brooks, 2013) and recurrent mutations in SKAP are correlated with aneuploidy in squamous cell carcinoma (Lee et al., 2014).

### Co-depletion of EB1 and EB3 delays the onset of anaphase

**Fig. 5.**
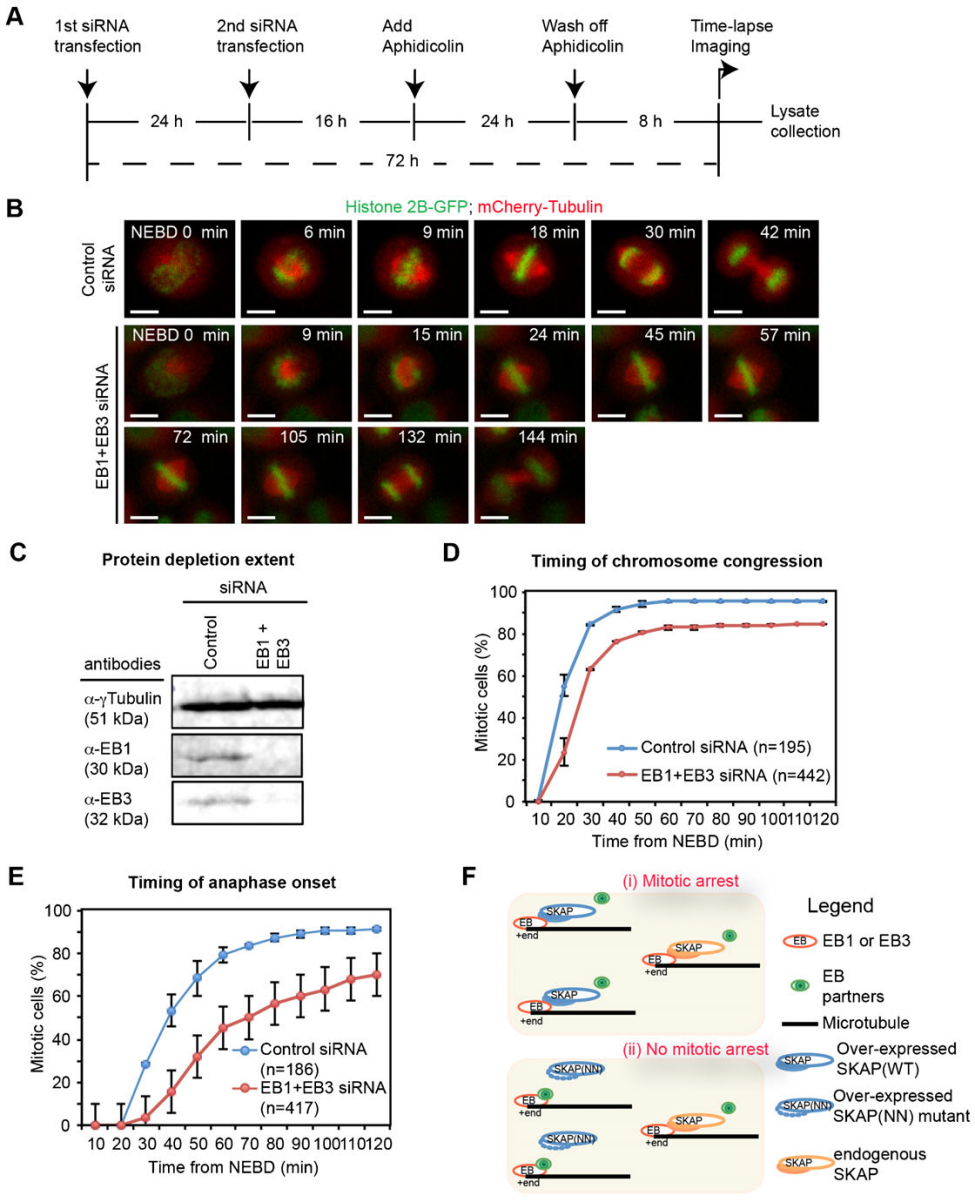
Co-depletion of EB1 and EB3 delays anaphase onset more severely than chromosome congression. (A) Schematic describing siRNA and aphidicolin treatments in HeLa^H2B-GFP mCherry-Tubulin^ cells. Cells were transfected with siRNA twice, at 72 h and 48 h prior to imaging. For synchronization, cells were treated with aphidicolin for 24 h, and then released into drug free medium for 8 h prior to imaging. At the end of imaging session, cell lysates were collected for immunoblotting to assess protein depletion extent. (B) Representative images from time-lapse movies of HeLa^H2B-GFP; mCherry-Tubulin^ cells treated with both EB1 and EB3 or Control siRNA oligos as in (A). Scale bar: 10 µm. (C) Representative immunoblots showing the co-depletion of EB1 and EB3 in lysates collected from time-lapse imaging studies as shown in (B). Lysates of HeLa^H2B-GFP; mCherry-Tubulin^ cells treated with indicated siRNA were processed for immunoblotting with α-γTubulin, α-EB1 and α-EB3 antibodies. (D-E) Cumulative frequency distribution graphs showing the time of chromosome congression (D) and anaphase onset (E) from NEBD in HeLa^H2B-GFP; mCherry-Tubulin^ cells treated with control alone or EB1 and EB3 siRNA oligos. The graphs present the average values of two independent experiments with the error bars showing SEM values. n refers to number of cells. (F) Cartoon illustrating the loss of EB binding with its partners in the presence of excess SKAP (i), but not SKAP (NN) mutant (ii) that is defective for EB interaction. Excess of SKAP-EB interaction results in mitotic arrest, and this model highlights the importance of regulated plus-end complex interactions for controlling the timing of anaphase onset.

Our data thus far shows that the abrogation of SKAP-EB interaction, in SKAP(NN) expressing cells, fully rescues SKAP overexpression induced delay in anaphase onset. Therefore, we hypothesized that excessive SKAP-EB interaction might lead to a loss of EB function and thus delay anaphase onset. If our hypothesis is correct, we would expect a similar anaphase delay following the loss of EB1 and EB3 proteins. In support of our hypothesis, a recent study indicated that the average anaphase onset is slightly reduced in cells co-depleted of EB1 and EB3 (Ferreira et al., 2013). However, it is not known whether anaphase onset delay is related to chromosome congression delay in cells depleted of EB1 and EB3 proteins. Therefore, we compared the rates of anaphase onset and completion of chromosome congression in EB1 and EB3 depleted HeLa^His2B-GFP; mCherry-Tubulin^ cells using time-lapse microscopy (Fig. 5A,B). To ensure that both EB1 and EB3 are fully depleted in our time-lapse microscopy studies, we harvested cell extracts at the end of each time-lapse imaging session and quantified the extent of protein depletion using fluorescent immunoblotting. In time-lapse movies of cell cultures that showed near-complete depletion of EB1 and EB3 (Fig. 5C), we quantified the time taken for two key events: (i) alignment of last chromosome on metaphase plate (from Nuclear Envelope Break-Down (NEBD) to completion of chromosome congression) and (ii) initiation of chromatid separation (from NEBD to initiation of anaphase onset). Our time-lapse analysis showed a pronounced anaphase onset delay in the vast majority of EB1 and EB3 co-depleted cells but not control-depleted cells. Importantly, comparing the rate and plateau in our timing graphs showed that the delay in anaphase onset was more pronounced compared to the delay in completing chromosome congression (compare Fig. 5D,E). This shows the importance of EB function in timing the onset of anaphase.

The pronounced anaphase delay in EB1 and EB3 co-depleted cells, compared to the noticeable but subtle delay in congression, further supports our model that excessive SKAP-EB interaction induced loss of EB function may be responsible for delaying anaphase without severely disrupting chromosome congression. Thus, the disruption of SKAP-EB interaction and the co-depletion of EB1 and EB3 result in similar mitotic outcomes. This correlative evidence further illustrates the general importance of EB and SXIP-motif mediated plus-end regulation in ensuring the normal timing of anaphase onset.

## DISCUSSION

To understand how microtubule-ends perform several spatially and temporally distinct tasks, we set out to obtain a comprehensive map of EB1 interactors from two distinct phases of mitosis. This proteome-wide study has unraveled several mitosis phase-specific interactors of EB1 and in addition revealed the Astrin/SKAP complex as a mitotic phase determined interactor of EB1. Investigating the significance of mitotic phase determined SKAP-EB interaction, revealed four unrecognised roles for the SKAP-EB interaction mediating SXIP motif. The SXIP motif controls (i) SKAP's interaction with EB3 (ii) SKAP's role in regulating MT growth, (iii) SKAP's localisation onto spindle microtubules and (iv) SKAP's role in controlling anaphase onset times. Thus regulated interaction of microtubule plus-end complexes may represent a key rate-limiting step in determining anaphase onset, independent of chromosome congression, and in turn defining mitotic outcome.

Mitotic phase-specific changes in phosphorylation of various microtubule-associated proteins are known (Pagliuca et al., 2011) but it is unclear how these phosphorylations change microtubule-end composition. Phosphorylation of the interphase plus-end binding proteins SLAIN2 (van der Vaart et al., 2011) and CLASP2 (Kumar et al., 2012) that directly interact with EB1, is known to dislodge them from plus-ends during mitosis. Expanding this knowledge, our effort provides a comprehensive list of EB1 interactions that change through mitotic phases. Interaction between CLASP2 and EB1 is likely to be spatially regulated because although CLASP2 is excluded from mitotic plus-ends (Kumar et al., 2012), CLASP2 is recruited to kinetochore-microtubule interface (Pereira et al., 2006) that is enriched for EB1 (Tirnauer et al., 2002), consistent with our finding of CLASP2 in EB1 immunoprecipitates from mitotic cells. Such refined spatial control over plus-end protein interactions during mitosis is probably achieved through localized kinase and phosphatase activities, a poorly understood area (reviewed in Tamura and Draviam, 2012).

Our mitotic-phase enrichment strategy has allowed high-throughput immunoprecipitation studies to reliably capture mitotic-phase associated changes in plus-end complexes. Previous SILAC-based quantitative *in vitro* approaches could distinguish microtubule-binding proteins from interphase *versus* mitotic cells (Syred et al., 2013). Thus, a SILAC-based methodology, in combination with our mitotic-phase enrichment strategy, should be a viable future option.

The outer kinetochore proteins, HEC1^Ndc80^ and KNL1^SPC105^ can directly contact the microtubule wall (Cheeseman et al., 2006; Wei et al., 2007). In addition, kinetochore bound motors and microtubule associated proteins can serve as additional molecular bridges between the microtubule and kinetochore (Hsu and Toda, 2011; Jeyaprakash et al., 2012; Maiato et al., 2003; Maure et al., 2011; Schmidt et al., 2012; Steuer et al., 1990; Wang et al., 2012; Wood et al., 1997). However, it is unclear how protein-protein interactions at the kinetochore-microtubule interface accommodate structural changes of the growing and shrinking phases of microtubule ends. Our finding that the SXIP-motif of SKAP is dispensable for its recruitment to kinetochores demonstrates that the kinetochore bound pool of SKAP does not require EB1 or EB3 interaction. This is consistent with the recruitment of SKAP to anaphase kinetochores (Dunsch et al., 2011; Fang et al., 2009; Schmidt et al., 2010) that are predominantly tethered to depolymerizing microtubule-ends lacking EB proteins. Thus, our molecular evidence for two pools of SKAP – one that requires regulated SXIP-motif interaction for proper microtubule growth and the other that associates with kinetochore in a SXIP-motif independent manner – reveals the Astrin-SKAP complex as a unique class of outer kinetochore bound microtubule associated protein that arrives at the kinetochores following microtubule-end association (Shrestha and Draviam, 2013) and remains at the kinetochore-microtubule interface regardless of structural changes associated with the presence or absence of microtubule growth associated EB proteins.

Despite SKAP's ability to directly interact with microtubules *in vitro*, independent of EB1 (Dunsch et al., 2011; Wang et al., 2012), we find that SKAP-EB interaction is essential for SKAP's localization onto spindle microtubules *in vivo*. SKAP-EB interaction must be finely regulated since we find that excess of SKAP-EB interaction disrupts mitotic timing and microtubule growth rates. We propose that SKAP-EB interaction in prometaphase must be regulated so that EB's interaction with other EB partners can remain unperturbed (Fig. 5F). In support of this model, the defects in microtubule growth and anaphase onset associated with SKAP overexpression are absent in cells overexpressing SKAP(NN) mutant (Fig. 5F) but are present in cells co-depleted of EB1 and EB3 proteins (Ferreira et al., 2013; this study).

Although, our MS studies could not recover any peptide bearing the SXIP motif of SKAP for confirming the phosphorylation status around SKAP's SXIP motif, we propose that SKAP-EB interaction may be temporally controlled by a mitotic phase-specific kinase or phosphatase for 3 reasons: (i) The proteomic data presented here show the interaction between the Astrin-SKAP complex and EB1 to be mitotic phase dependent. (ii) Our protein-protein interaction studies show that phospho-modulation of an Aurora-B consensus site near the SXIP motif is sufficient for negatively regulating SKAP-EB interaction. (iii) In *S. cerevisiae*, phosphorylation near the SXIP-motif (Zimniak et al., 2009) is reported to control EB1/Bim1 interaction with AuroraB/Ipl1 in anaphase.

Throughout this study, we recurrently found evidence for spatially and temporally regulated interaction among plus-end proteins during mitosis: in our proteomic studies of mitotic phase-specific EB1 interactors, in our localisation studies of SKAP-EB interaction dependent SKAP enrichment on spindle microtubules, and in our time-lapse studies of SKAP-EB interaction dependent changes in microtubule growth and anaphase onset. Thus, determining mitotic phase-specific EB interactions is a crucial step towards our understanding of how microtubule plus-end complexes execute spatially and temporally distinct mitotic events.

## MATERIALS AND METHODS

### Cell culture and synchronization

UTA6 and HeLa cells were cultured in Dulbecco's modified Eagle's medium supplemented with 10% FCS and antibiotics, penicillin and streptomycin. Cells were plated onto plastic dishes for large-scale cell culture and glass-bottomed dishes (LabTek) or 13 mm round coverslips for imaging. For inhibition studies, cells were treated with 10 µM MG132 (1748, TOCRIS). Double-thymidine block for synchronisation was performed on 60% confluent cell cultures by treating cells with 2 mM thymidine (ACROS organics) for 24 h, releasing cells from the thymidine treatment for 12 h, treating cells with a second round of 2 mM thymidine for 12 h and finally releasing them for thymidine treatment for 9–10 h for mitotic cell enrichment. For large-scale prometaphase cell enrichment, soon after the second round of thymidine release, 5 µM DMA was added (Enzo Life Sciences) and 14 h later rounded up cells were collected by shake-off. For anaphase cell enrichment, prometaphase cells were washed with warm DMEM and released into drug free medium for 45 min.

### Large-scale immunoprecipitation

60% confluent UTA6 cell cultures, in 20 large (15 cm) plates, grown in the presence of Tetracycline were synchronized using double-thymidine blocks and DMA treatment (14 h), and harvested by shake-off 24 h after Tetracycline release. Cell pellets were suspended in 5 ml of lysis buffer (50 mM Tris-Cl (pH 7.4–7.8), 150 mM NaCl, 2 mM EGTA, 1 mM EDTA, 0.1% (w/v) CHAPs, 0.1% Triton X-100, 3 mM NaF, 1 mM Na_3_VO_4_), mixed at 4°C for 20 min for further cell lysis. The cell lysate suspension was centrifuged (1000 rpm for 5 min) and the supernatant was collected for immunoprecipitation. For total lysate input control, 75 µl of the suspension was mixed with 4×SDS sample buffer and stored separately. For immunoprecipitation anti-mouse Flag antibody (Sigma-Aldrich; F3165) was added and then rotated at 4°C for 1 h. Dynabeads Protein G (250 µl of magnetic dynabeads that can absorb 50–75 µg of antibody) was added into the antibody containing cell suspension and this mix was then rotated at 4°C for 1 h. The tube was placed on the magnetic stand and 75 µl of suspension was collected and mixed with 4×SDS sample buffer as the supernatant (unbound fraction). The rest of the suspension was removed from the tube and unspecific proteins on the beads were removed by five rounds of washes that included adding 1 ml of wash buffer [3 mM NaF, 1 mM Na3VO_4_, 0.05% (w/v) Triton, 1× Protease inhibitor cocktail (Roche) in PBS (PAA)], rotating the tube at 4°C for 3 min, placing tube on magnetic stand and discarding the supernatant. Subsequently the immunoprecipitated protein complexes were eluted with anti-Flag peptide containing elution buffer (50 µl; anti-3×Flag peptide (1 mg/ml) (Invitrogen F4299), 3 mM NaF, 1 mM Na_3_VO_4_, 0.05% (w/v) Triton, 1× Protease inhibitor cocktail) by vortexing for 20 min at 4°C and then the elutant was transferred into a new tube. This elution step was repeated 5 times and the elutant and beads were stored at −20°C. To test the efficiency 10% of elutant was mixed with 4×SDS buffer and loaded onto a gel for immunoblotting. The remaining elutant was precipitated with acetone (1:10 elutant: acetone) for 20 min at −20°C and centrifuged (6000 rpm, 10 min), supernatant was removed and pellet dried at room temperature for 10 min. The dry pellets were processed for mass spectrometry analysis at the Institute of Biochemistry and Biophysics in the Polish Academy of Science. For one of the repeats, the mass spectrometry analysis was performed at the Cambridge Centre for Proteomics, University of Cambridge.

### Yeast two hybrid (Y2H) analysis

Human cDNA fragments encoding for EB1 (NM_012325), EB3 (NM_012326), SKAP (NM_033286.2), Astrin (NM006461.3) were subcloned into pGBT9 and pGAD424 (Clontech). SKAP point mutants (^113^LP to NN and RAT^108^ into either RAA or RAE) were created by PCR mutagenesis and confirmed by DNA sequencing. Yeast two-hybrid protocols were based on the Matchmaker 3 yeast two-hybrid system (Clontech).

### Yeast strains and plasmids used in this study

Yeast strains (AH109 and Y187) and plasmid vectors (pGAD242 and pGBT9) were kindly provided by V Bolanos Garcia (Blundell group, University of Cambridge). N-terminally tagged Flag-EB1 or Flag-Nuf2 were generated using the Tetracycline-repressible vector pTRE-Tight-BI-AcGFP1 (Clontech). N-terminally tagged GFP-SKAP was generated using the Tetracycline-inducible FRT/TO system (Life Technologies).

### Live-cell studies (time-lapse imaging)

Cells were transfected with siRNA oligos or plasmid vectors for 48 or 24 h, respectively, prior to imaging and transferred to Leibovitz's L15 medium (Invitrogen) for imaging at 37°C. For live-cell imaging movies of GFP-SKAP(WT) or GFP-SKAP(NN) expressing cells, exposures of 0.02–0.05 s was used for acquiring three Z-planes, 3 µm apart, once every 3 min for 6 to 8 h, with a 40 times NA 0.75 objective. For live-cell imaging movies of EB3-mKate comets, exposures of less than 0.02 s was used for acquiring at least 10 Z-planes, 0.1 µm apart, in continuous acquisition mode, for 5 min, with a 100× NA 1.4 objective. All live-cell imaging studies were performed on an Applied Precision DeltaVision Core microscope equipped with a Cascade2 camera under EM mode.

### siRNA transfection

The following published siRNA oligos were used: EB1 oligo (5′-UUGCCUUGAAGAAAGUGAA dT.dT-3′; Dharmacon) (Draviam et al., 2006) and EB3 oligo (5′-CCAUGAGACUGAUGCCCAAAUUCUU-3′; Invitrogen) (Ban et al., 2009). siRNA transfection were carried out twice, 48 h and 72 h prior to imaging, using Oligofectamine (Life Technologies) according to manufacturer's instruction.

### Immunofluorescence and immunoblotting

For immunofluorescence, antibodies against Tubulin (Abcam; ab6160), Flag (Sigma; F7425), Tubulin (Sigma; T4026), GFP (Roche; 1181446001), SKAP (Atlas; HPA042027), and CREST anti-sera (Europa; FZ90C-CS1058) were used. Images of immunostained cells were acquired using 100 times NA 1.4 objective on a DeltaVision Core microscope equipped with CoolSnap HQ Camera (Photometrics). For immunoblotting, antibodies against Tubulin (Sigma-Aldrich; T6557), EB1 (BD; 610534), flag (Sigma; F3165), EB3 (Millipore; AB6033), Cyclin-B (BD; 554176) were used. Immunoblots were developed using fluorescent secondary antibodies (LI-COR) and fluorescent immunoblots were quantified using the Odyssey (LI-COR) software. For allowing the merging of images from two different fluorescent channels of a single blot as in Fig. 1H, mouse anti-flag and rabbit anti-SKAP antibodies were used.

### Mass spectrometry analysis

Peptides mixtures were analyzed by LC-MS-MS/MS (liquid chromatography coupled to tandem mass spectrometry) using Nano-Acquity (Waters) LC system and Orbitrap Velos mass spectrometer (Thermo Electron Corp., San Jose, CA). Prior to the analysis, proteins were subjected to standard “in-solution digestion” procedure during which proteins were reduced with 100 mM DTT (for 30 min at 56°C), alkylated with 0.5 M iodoacetamide (45 min in a darkroom at room temperature) and digested overnight with trypsin (sequencing Grade Modified Trypsin – Promega V5111). Peptide mixture was applied to RP-18 precolumn (nanoACQUITY Symmetry® C18 – Waters 186003514) using water containing 0.1% TFA as mobile phase and then transferred to nano-HPLC RP-18 column (nanoACQUITY BEH C18 – Waters 186003545) using an acetonitrile gradient (0%-60% AcN in 120 min) in the presence of 0.05% formic acid with the flowrate of 150 nl/min. Column outlet was directly coupled to the ion source of the spectrometer working in the regime of data dependent MS to MS/MS switch. A blank run ensuring lack of cross contamination from previous samples preceded each analysis. Acquired raw data were processed by Mascot Distiller followed by Mascot Search (Matrix Science, London, on-site license) against SwissProt database. Search parameters for precursor and product ion mass tolerance were 20 ppm and 0.6 Da, respectively, with search parameters set as follows: one missed semiTrypsin cleavage site allowed, fixed modification of cysteine by carbamidomethylation and variable modification of lysine carbamidomethylation and methionine oxidation. Peptides with Mascot score exceeding the threshold value corresponding to <5% false positive rate, calculated by Mascot, were considered to be positively identified.

### Statistical analysis

Error bars indicate SD or SEM values obtained across experiments or cells as indicated in legend. p-values representing significance were obtained using Proportion test on percentage values. To produce histogram and to analyse Wilcoxon rank sum test, we used R 3.0.2 GUI 1.62 Snow Leopard build.

## Supplementary Material

Supplementary Material
