# Supplementary material for: A proteomic study of mitotic phase-specific interactors of EB1 reveals a role for SXIP-mediated protein interactions in anaphase onset
Source: Biol Open. 2015 Jan 16;4(2):155–69. doi: 10.1242/bio.201410413 (PMC4365484; doi:10.1242/bio.201410413)
Supplement: Supplementary Material [file supp_4_2_155__index.html]

A proteomic study of mitotic phase-specific interactors of EB1 reveals a role for SXIP-mediated protein interactions in anaphase onset — A proteomic study of mitotic phase-specific interactors of EB1 reveals a role for SXIP-mediated protein interactions in anaphase onset — Supplementary Material 

# A proteomic study of mitotic phase-specific interactors of EB1 reveals a role for SXIP-mediated protein interactions in anaphase onset

## bio.201410413 Supplementary Material

**Files in this Data Supplement:**

- Supplementary Material - Naoka Tamura et al. doi: 10.1242/bio.201410413
