# Supplementary material for: A proteomic study of mitotic phase-specific interactors of EB1 reveals a role for SXIP-mediated protein interactions in anaphase onset
Source: Biol Open. 2015 Jan 16;4(2):155–69. doi: 10.1242/bio.201410413 (PMC4365484; doi:10.1242/bio.201410413)
Supplement: Supplementary Material [file supp_bio.201410413_bio.201410413-s1.pdf]

Biology Open (2015) 000, 1–15 doi:10.1242/bio.201410413

Supplementary Material

Naoka Tamura et al. doi: 10.1242/bio.201410413

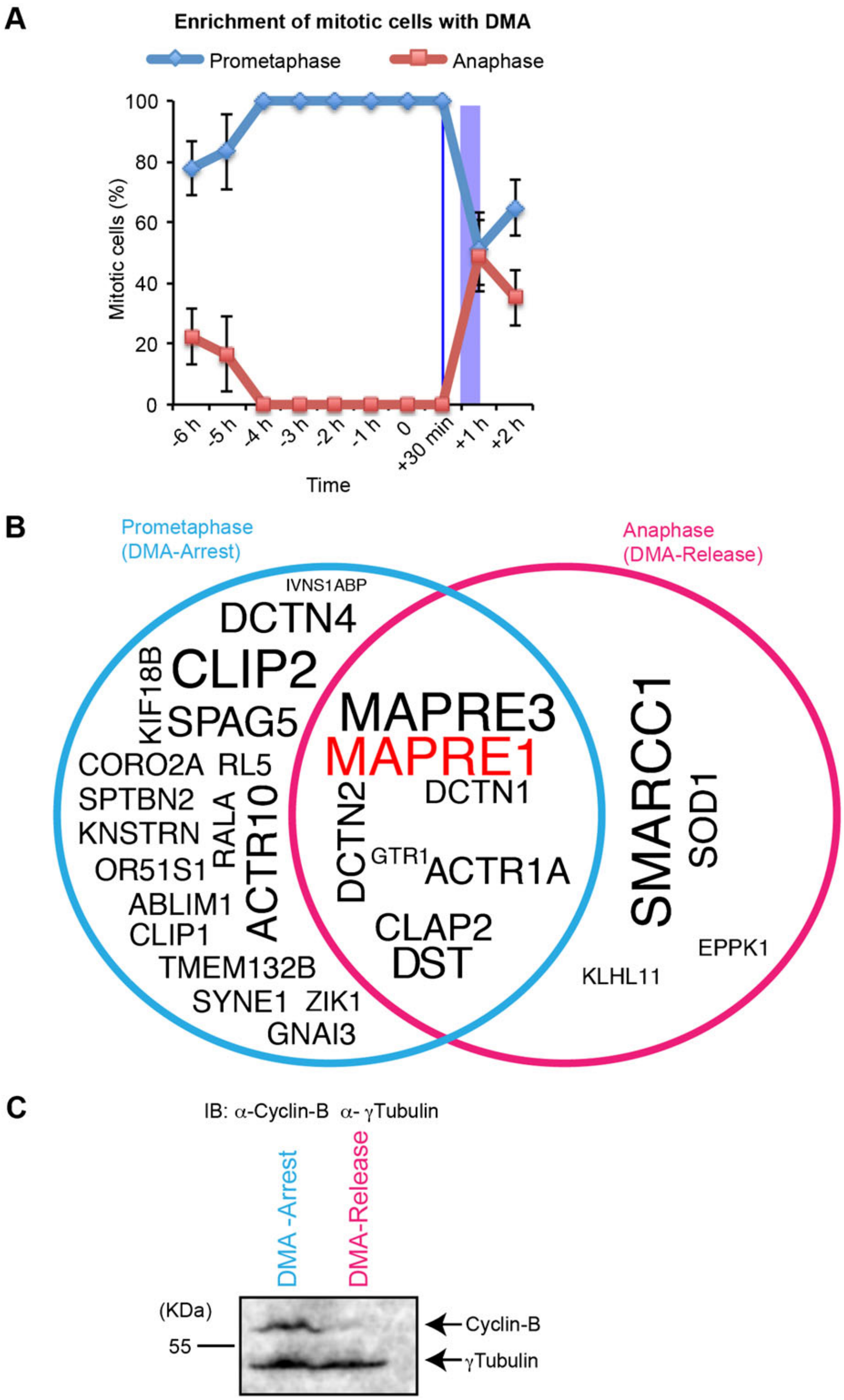

Fig. S1. Mitotic phase-specific enrichment of EB1 interactors. (A) Graph showing the percentage of cells in distinct mitotic phases following DMA treatment (DMA-arrest) and release from DMA treatment (DMA-release). In plastic dishes UTA6 cells were cultured and prometaphase (Prometa) and anaphase (Ana) cells were counted by DIC microscopy at each time point (n=300 cells). Mitotic cells (Mito) represent a total number of 'rounded-up' prometaphase cells and 'elongated' anaphase cells. Cells were synchronized by double thymidine blocks and treated using DMA (5 μM) for 6 h. DMA was removed by serial-washing and the cells were released into drug free DMEM media for 2 h. Vertical blue line represents time-point for shaking-off prometaphase cells for large-scale immunoprecipitations. Vertical box in purple refers to harvesting time-point for anaphase cells (a box is chosen as the time period includes five brief washes of three minute centrifuge-runs to thoroughly wash-off DMA, which was followed by a 45 minute DMA-free incubation period). On the X-axis, prometaphase harvesting time-point as T=0. Note that the percentages were derived from non-shaken-off cell cultures on plastic dishes, hence is an under-estimate of mitotic enrichment efficiency. (B) Venn diagram showing EB1 interactors in DMA-arrested (Blue circle), -released (Pink circle) or both (overlapped region) immunoprecipitations. Compared to Fig. 1F, this diagram presents a high stringency cut-off to isolate EB1 specific interactors. Diagram excludes proteins found even once in any of the Flag-Nuf2 IPs and includes proteins found in Flag-EB1 IPs at least twice. Font size in each area of the circle reflects reproducibility across repeats. Bait is highlighted red. (C) Immunoblots showing reduction in Cyclin-B levels in lysates of prometaphase (DMA-arrest) compared to anaphase (DMA-release) UTA6 Flag-EB1 cells. Immunoblots were probed with α-CyclinB1 and α-γTubulin antibodies. Lysates are from a batch-matched experiment shown in Fig. 2A.

Biology Open (2015) 000, 1–15 doi:10.1242/bio.201410413

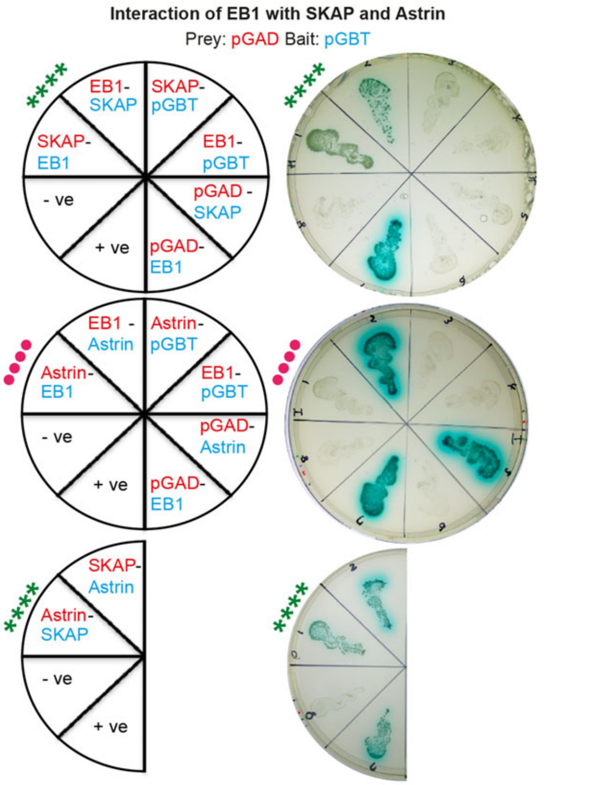

Fig. S2. Yeast two hybrid study showing the lack of interaction between EB1 and Astrin. Yeast strains carrying plasmids encoding EB1, SKAP or Astrin are indicated on pie chart (left). Positive interaction was assessed through the activation of the *lacZ* reporter gene which was demonstrated by the formation of blue colonies on plates containing X-Gal. Photographs of colonies (right) show a positive interaction between EB1 and SKAP or SKAP and Astrin (blue colour development; marked **** in green) and absence of interaction between EB1 and Astrin (no blue colour development; marked •••• in red). The interaction of SV40-p53 and SV40-Laminin were used as positive (+ve) and negative (-ve) controls, respectively.

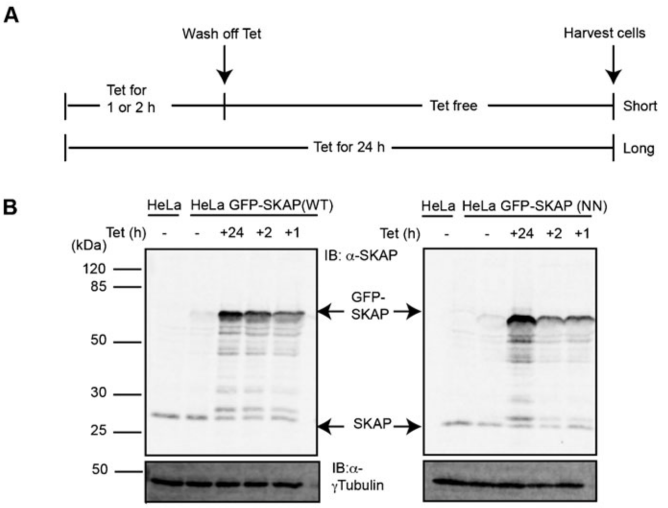

Fig. S3. GFP-tagged SKAP (WT) and SKAP (NN) mutant protein expression levels are comparable and not different. (A) Schematic describing Tetracycline induction in HeLa$^{GFP\text{-}SKAP(WT)}$ or HeLa$^{GFP\text{-}SKAP(NN)}$ cell line. Cells were treated with Tetracycline (Tet) for a long period (24 h), or short period (2 or 1 h). For short treatments, Tet was washed off and the cells were maintained in Tet-free media for up to 24 h. (B) Immunoblots showing similar levels of GFP-SKAP(WT) or GFP-SKAP(NN) in lysates of cells treated with Tetracycline (Tet) as illustrated in (A). Lysates were subjected to immunoblotting using α-SKAP and α-γTubulin antibodies. γTubulin was loaded as control. Endogenous SKAP and GFP-tagged SKAP are marked with arrows. HeLa cell lysates were loaded as control showing the absence of GFP-SKAP.
